# Abnormal whole-body energy metabolism in iron-deficient humans despite preserved skeletal muscle oxidative phosphorylation

**DOI:** 10.1038/s41598-021-03968-4

**Published:** 2022-01-19

**Authors:** Matthew C. Frise, David A. Holdsworth, Andrew W. Johnson, Yu Jin Chung, M. Kate Curtis, Pete J. Cox, Kieran Clarke, Damian J. Tyler, David J. Roberts, Peter J. Ratcliffe, Keith L. Dorrington, Peter A. Robbins

**Affiliations:** 1 Department of Physiology, Anatomy and Genetics, University of Oxford, Sherrington Building, Parks Road, Oxford, OX1 3PT UK; 2 Nuffield Department of Clinical Neurosciences, University of Oxford, John Radcliffe Hospital, Oxford, OX3 9DU UK; 3 Nuffield Department of Clinical Laboratory Sciences, National Blood Service Oxford Centre, University of Oxford, John Radcliffe Hospital, Oxford, OX3 9BQ UK; 4 Nuffield Department of Medicine, University of Oxford, NDM Research Building, Old Road Campus, Headington, Oxford, OX3 7FZ UK; 5 Francis Crick Institute, London, NW1 1AT UK

**Keywords:** Energy metabolism, Respiration, Mitochondria, Skeletal muscle

## Abstract

Iron deficiency impairs skeletal muscle metabolism. The underlying mechanisms are incompletely characterised, but animal and human experiments suggest the involvement of signalling pathways co-dependent upon oxygen and iron availability, including the pathway associated with hypoxia-inducible factor (HIF). We performed a prospective, case–control, clinical physiology study to explore the effects of iron deficiency on human metabolism, using exercise as a stressor. Thirteen iron-deficient (ID) individuals and thirteen iron-replete (IR) control participants each underwent ^31^P-magnetic resonance spectroscopy of exercising calf muscle to investigate differences in oxidative phosphorylation, followed by whole-body cardiopulmonary exercise testing. Thereafter, individuals were given an intravenous (IV) infusion, randomised to either iron or saline, and the assessments repeated ~ 1 week later. Neither baseline iron status nor IV iron significantly influenced high-energy phosphate metabolism. During submaximal cardiopulmonary exercise, the rate of decline in blood lactate concentration was diminished in the ID group (P = 0.005). Intravenous iron corrected this abnormality. Furthermore, IV iron increased lactate threshold during maximal cardiopulmonary exercise by ~ 10%, regardless of baseline iron status. These findings demonstrate abnormal whole-body energy metabolism in iron-deficient but otherwise healthy humans. Iron deficiency promotes a more glycolytic phenotype without having a detectable effect on mitochondrial bioenergetics.

## Introduction

Skeletal muscle metabolism is responsible for as much as 25% of resting oxygen consumption ($\dot {\rm V}$o_2_) in healthy young adult humans, and $\dot {\rm V}$o_2_ may increase 10–15 times above resting values during whole body exercise, even in untrained individuals^1^. Exercise thus places very considerable demands on those physiological processes concerned with pulmonary uptake of oxygen, its delivery to tissues, and its ultimate fate as the terminal electron acceptor in mitochondrial oxidative phosphorylation. Importantly, iron has indispensable roles in each of these processes.

Many previous studies have addressed the impact of iron deficiency on human exercise capacity. For example, in subjects with profound iron-deficiency anaemia (IDA), maximal treadmill exercise time increased significantly with intravenous (IV) iron supplementation, and post-exercise venous blood lactate concentrations remained similar with successive experiments despite higher workloads^2^. In iron-deficient (ID) female athletes, whilst oral iron supplementation had no effect on maximal workload on a bicycle ergometer, maximal blood lactate levels decreased following iron administration^3^.

However, there are major difficulties in interpreting the findings of such studies, which have almost invariably been performed in athletic individuals. Habitual exercise brings about a complex interplay of inflammatory and hypoxic stresses leading to alterations in the master iron-regulatory hormone hepcidin^4^. The result is that the pathophysiology of iron deficiency in endurance athletes may differ significantly from that of absolute iron deficiency in non-athletes^5^. Moreover, the use of oral iron over a protracted period inevitably leads to a rise in haemoglobin (Hb) concentration, which is a powerful confounding factor.

The challenge of separating the effects of iron deficiency from the consequences of coexisting anaemia has long been recognised^6,7^. Although a meta-analysis previously reported iron supplementation to be beneficial for improving maximal oxygen consumption ($\dot {\rm V}$o_2_max) in iron-deficient non-anaemic athletes^8^, iron supplementation predictably increased Hb concentration in the studies included. Given the strong correlation between red cell mass and $\dot {\rm V}$o_2_max in health, iron supplementation will inevitably increase $\dot {\rm V}$o_2_max over time by promoting a rise in Hb concentration. In a contemporary study where IV iron was administered to elite athletes without precipitating a rise in Hb concentration, no effect on $\dot {\rm V}$o_2_max was seen^9^.

It is possible to limit the confounding effects of Hb concentration using animal models. Isovolaemic transfusion experiments in rodents indicate that iron deficiency: (i) compromises work capacity during whole-body exercise^10^; (ii) promotes excessive blood lactate accumulation^11^; and (iii) leads to deranged skeletal muscle oxidative phosphorylation^12^. Whilst these and other studies^7,13,14^ indicate that IDA limits oxygen delivery to exercising muscle, they leave unanswered the question of how tissue iron deficiency per se disrupts oxidative metabolism.

A remarkably similar metabolic phenotype is evident in humans with a rare congenital disease of oxygen sensing, Chuvash polycythaemia (CP)^15^. Adult humans with CP show not only the diminished aerobic exercise capacity and hyperlactataemia of ID rodents, but also similarly disturbed skeletal muscle oxidative phosphorylation. Many of the features of CP result from dysregulation of the hypoxia-inducible factor (HIF) transcription factor pathway^16^, which regulates integrated physiological responses to changes in oxygen availability, including erythropoietin expression^17^. The oxygen sensors are HIF-hydroxylase enzymes, which utilise a single ferrous ion coordinated at their active sites. These enzymes hydroxylate the HIF-α subunit at either of two prolyl residues, targeting it for proteasomal degradation, or at an asparaginyl residue, reducing its interaction with a transcriptional coactivator protein^18^. As would be anticipated, iron availability thus plays a critical role in modulating physiological responses to hypoxia^19^.

Taking all this together, we hypothesised that clinical iron deficiency would disturb human metabolism in a manner similar to that seen both in rodent models of iron deficiency and in patients with CP. The present study compared healthy iron-replete (IR) participants with their ID – but not frankly anaemic – counterparts. Using an established approach^15^, we adopted a design involving: (i) a case–control approach for baseline comparisons, employing combined assessment of whole-body cardiopulmonary physiology and small muscle mass high-energy phosphate metabolism; and (ii) repeated assessment a short interval after randomisation to IV iron or saline control, before any change in Hb concentration could occur.

## Results

### Baseline characteristics of iron-deficient and iron-replete groups

Baseline characteristics of the participants are given in Table 1. Mean serum ferritin in the ID group was 8.3 μg/L, and transferrin saturation (TSat) 10.6%; plasma soluble transferrin receptor (sTfR) values in the ID group were approximately twice those of IR participants, and hepcidin, the major hormone regulating iron uptake and distribution, was very heavily suppressed in the ID group. These values confirm absolute iron deficiency^20^.

**Table 1:** Participant characteristics on enrolment.

Characteristic	ID Group (n = 13)	IR Group (n = 13)	P-value
Sex, M:F	1:12	1:12	
Age, years	23 (21.5–38)	24 (22–25.5)	0.59
BMI, kg/m^2^	21.8 (20.8–24.1)	21.1 (20.7–24.1)	1.0
Resting S_p_o_2_, %	98.8 ± 0.9	98.5 ± 1.1	0.45
Systolic BP, mmHg	119 ± 11	119 ± 9	0.99
Diastolic BP, mmHg	73 ± 10	79 ± 8	0.08
FEV_1_, % predicted	106 ± 12	104 ± 9	0.75
Exercise, h/week	5.0 (2.0–7.5)	4.0 (1.0–8.5)	0.70
Plasma CRP, mg/L	0.4 (0.3–2.6)	0.5 (0.3–1.6)	0.96
Serum ferritin, μg/L	8.3 ± 3.1	58.0 ± 38.2	N/A
Serum TSat, %	10.6 ± 3.9	35.2 ± 7.6	N/A
Serum iron, μmol/L	8.4 ± 3.2	22.0 ± 3.6	**< 0.001**
Serum transferrin, g/L	3.6 ± 0.6	2.9 ± 0.5	**0.003**
Plasma sTfR, nmol/L	38.4 (32.7–50.9)	21.8 (18.4–25.0)	**< 0.001**
Plasma hepcidin, μg/L	2.0 (1.3–2.4)	13.9 (9.9–29.5)	**< 0.001**
Haemoglobin, g/dl	12.7 (11.5–13.3)	13.6 (13.5–14.0)	**< 0.001**
Haematocrit, %	39.7 ± 3.2	42.0 ± 1.7	**0.028**
Mean cell volume, fl	86.9 (83.0–91.3)	92.4 (89.9–94.1)	**0.010**

For normally distributed data, comparisons are by t-test and values are means ± SD. For non-normally distributed data, comparisons are by Mann Whitney U test and values are medians (IQR). Statistically significant P-values appear in bold. ID, iron deficient; IR, iron replete; BMI, body mass index; S_p_o_2_, peripheral oxyhaemoglobin saturation; FEV_1_, forced expiratory volume in one second; CRP, C-reactive protein; TSat, transferrin saturation; sTfR, soluble transferrin receptor.

### Effect of iron infusion on laboratory parameters at the second study visit

Table 2 shows the changes in laboratory parameters between study visits, which were conducted approximately a week apart. There was no significant change in Hb concentration between study visits. Participants randomised to receive iron showed the expected significant increases in ferritin, serum iron, TSat and hepcidin; conversely, transferrin and sTfR fell significantly, also as expected. However, although the direction of effect following IV administration of iron was the same in ID and IR groups for each of these parameters, ID subjects did not attain the same absolute values in in any of the indices compared with IR subjects. Iron administration influenced circulating erythropoietin levels in ID participants, but not IR controls (P = 0.015 for interaction between study visit, baseline iron status and intervention).

**Table 2:** Haematological and iron parameters over the course of the study.

Iron status	Iron deficient (n = 13)				Iron replete (n = 13)				P-value for interactions (RM-ANOVA)	
Intervention	Iron (n = 7)		Saline (n = 6)		Iron (n = 7)		Saline (n = 6)		Visit & intervention	Iron status, visit & intervention
Visit	1	2	1	2	1	2	1	2		
Hb, g/dl	13.1 ± 1.1	13.0 ± 1.3	12.7 ± 0.6	12.5 ± 0.7	14.3 ± 0.7	13.7 ± 0.9	14.1 ± 0.4	13.9 ± 0.6	0.66	0.43
MCV, fl	84.4 ± 7.6	87.1 ± 6.8	86.0 ± 3.9	86.0 ± 4.3	91.9 ± 5.7	91.4 ± 2.6	90.9 ± 3.2	91.3 ± 2.3	0.47	0.09
Serum ferritin, μg/L	7.9 ± 3.5	561 ± 433	6.9 ± 2.4	10.6 ± 2.8	56.3 ± 35.5	840 ± 331	29.0 ± 14.3	38.7 ± 16.2	**< 0.001**	0.32
Serum TSat, %	12.3 ± 4.9	37.9 ± 11.2	13.7 ± 5.5	11.0 ± 2.8	29.0 ± 8.8	62.1 ± 20.1	23.0 ± 5.5	24.3 ± 12.5	**< 0.001**	0.73
Serum iron, μmol/L	9.3 ± 2.6	24.0 ± 9.1	11.1 ± 4.8	8.7 ± 2.0	17.8 ± 4.8	31.2 ± 10.4	16.2 ± 4.3	16.5 ± 8.2	**< 0.001**	0.54
Serum transferrin, g/L	3.61 ± 0.77	2.90 ± 0.48	3.65 ± 0.49	3.68 ± 0.44	2.83 ± 0.34	2.32 ± 0.26	3.17 ± 0.40	3.13 ± 0.39	**< 0.001**	0.27
Plasma sTfR, nmol/L	40.3 ± 12.0	33.4 ± 7.6	41.8 ± 13.1	40.9 ± 14.0	24.3 ± 3.5	20.7 ± 4.2	22.5 ± 2.5	21.7 ± 2.0	**0.007**	0.30
Serum Epo, mIU/ml	17.7 ± 9.2	13.6 ± 3.9	13.8 ± 4.7	21.3 ± 7.8	8.0 ± 2.8	7.6 ± 4.0	7.7 ± 2.4	7.2 ± 2.1	**0.016**	**0.015**
Plasma hepcidin, μg/L	2.1 ± 1.2	38.5 ± 28.9	1.5 ± 0.5	2.1 ± 1.0	17.2 ± 22.6	85.5 ± 54.6	7.3 ± 3.3	14.6 ± 14.7	**< 0.001**	0.30

Values are means ± SD. Statistically significant P-values appear in bold. RM-ANOVA, repeated measures analysis of variance; MCV, mean cell volume; TSat, transferrin saturation; sTfR, soluble transferrin receptor; Epo, erythropoietin.

### Skeletal muscle oxidative phosphorylation

**Figure 1:**
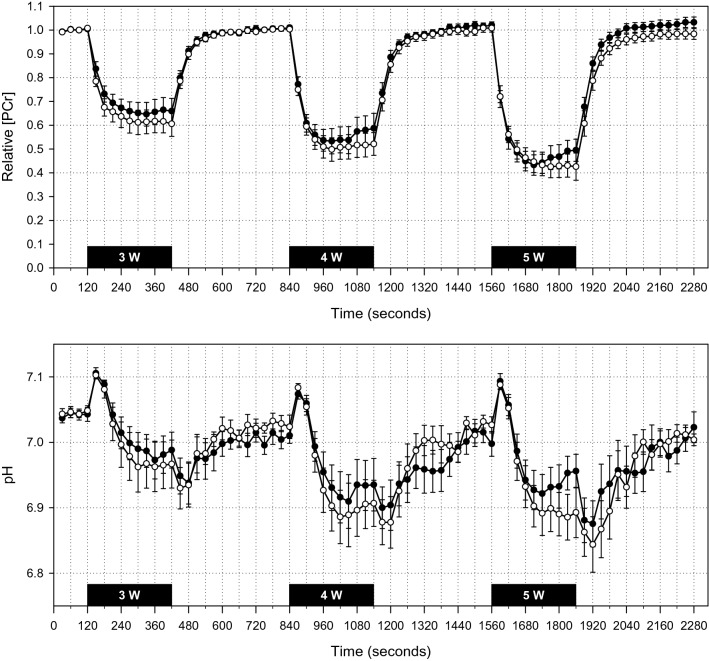
^31^P-MRS data from first study visit. [PCr] is expressed as a fraction of the mean value during the initial 2-min rest period. Data for ID participants appear as white circles; those for IR participants, black circles. Solid black bars indicate 5-min exercise periods. All participants completed the 3-W exercise bout. Subsequently, several participants ceased exercise prematurely due to fatigue: during the 4-W bout, one IR participant after 1050 s; during the 5-W bout, one ID participant after 1820s, one IR participant after 1720s, and another IR participant after 1775s. For illustrative purposes, recovery data for these participants are shifted to align with cessation of exercise in the other participants; an ‘early recovery’ artefact is thus apparent in the IR group near the end of the 4-W and 5-W bouts. The rest periods include data for all participants. Values are 30-s means; error bars show SE.

High-energy phosphate metabolism and intracellular pH changes during the first study visit are illustrated in Fig. 1. An analysis of the effect of iron status, workload, and iron infusion based on monoexponential fitting of data from the recovery period after each bout of exercise is given in Table 3. The only significant effect detected was of workload, with end-exercise PCr and pH tending to fall with successive bouts of small-muscle-mass exercise, both as expected. No effect was evident of either baseline iron status or IV iron administration.

**Table 3:** Parameters derived from monoexponential fitting of PCr recovery data.

Parameter	Iron status	Iron deficient (n = 13)				Iron replete (n = 13)				P-value (RM-ANOVA)
	Intervention	Iron (n = 7)		Saline (n = 6)		Iron (n = 7)		Saline (n = 6)		
	Visit	1	2	1	2	1	2	1	2	
[PCr]	3 W	0.72 ± 0.11	0.74 ± 0.10	0.57 ± 0.15	0.62 ± 0.23	0.74 ± 0.10	0.73 ± 0.09	0.60 ± 0.12	0.53 ± 0.19	Iron status = 0.99 **Workload < 0.001** Visit & intervention = 0.60 Iron status, visit & intervention = 0.48
	4 W	0.59 ± 0.15	0.61 ± 0.18	0.45 ± 0.17	0.47 ± 0.22	0.68 ± 0.10	0.63 ± 0.12	0.44 ± 0.19	0.44 ± 0.22	
	5 W	0.50 ± 0.20	0.52 ± 0.21	0.38 ± 0.19	0.38 ± 0.24	0.50 ± 0.14	0.50 ± 0.18	0.44 ± 0.20	0.32 ± 0.23	
pH	3 W	6.99 ± 0.13	7.00 ± 0.06	6.95 ± 0.16	6.91 ± 0.25	7.04 ± 0.03	7.04 ± 0.04	6.92 ± 0.11	6.98 ± 0.10	Iron status = 0.75 **Workload < 0.001** Visit & intervention = 0.90 Iron status, visit & intervention = 0.37
	4 W	6.92 ± 0.11	6.94 ± 0.11	6.90 ± 0.15	6.86 ± 0.24	6.98 ± 0.10	6.97 ± 0.11	6.86 ± 0.15	6.88 ± 0.07	
	5 W	6.90 ± 0.09	6.90 ± 0.14	6.88 ± 0.18	6.87 ± 0.24	6.95 ± 0.11	6.90 ± 0.14	6.87 ± 0.14	6.86 ± 0.09	
τ	3 W	35 ± 14	32 ± 4	40 ± 14	51 ± 29	32 ± 16	29 ± 8	43 ± 16	49 ± 23	Iron status = 0.79 Workload = 0.16 Visit & intervention = 0.14 Iron status, visit & intervention = 0.34
	4 W	36 ± 13	35 ± 6	41 ± 15	52 ± 31	43 ± 16	33 ± 11	43 ± 16	46 ± 15	
	5 W	40 ± 15	35 ± 9	45 ± 19	50 ± 19	45 ± 15	41 ± 14	45 ± 15	47 ± 19	
Qmax	3 W	0.33 ± 0.08	0.37 ± 0.18	0.41 ± 0.17	0.25 ± 0.07	0.41 ± 0.24	0.44 ± 0.17	0.31 ± 0.03	0.49 ± 0.37	Iron status = 0.32 **Workload < 0.001** Visit & intervention = 0.16 Iron status, visit & intervention = 0.08
	4 W	0.43 ± 0.16	0.44 ± 0.29	0.48 ± 0.16	0.42 ± 0.32	0.47 ± 0.18	0.48 ± 0.12	0.43 ± 0.11	0.54 ± 0.28	
	5 W	0.43 ± 0.09	0.51 ± 0.24	0.46 ± 0.12	0.36 ± 0.07	0.53 ± 0.11	0.52 ± 0.18	0.38 ± 0.09	0.59 ± 0.30	

Values are given at three different workloads (3 W, 4 W and 5 W) for: (i) [PCr], phosphocreatine concentration at end exercise, expressed as a fraction of the resting level to which it subsequently recovered; (ii) pH at end exercise; (iii) τ, measured in seconds; and (iv) Qmax, measured in mM of ATP per second. For individuals stopping work prematurely owing to fatigue, recovery kinetics were modelled from cessation of exercise. Values are means ± SD. Statistically significant P-values appear in bold.

### Exercise to volitional fatigue

Table 4 shows physiological parameters at the point of volitional fatigue during maximal cardiopulmonary exercise testing (CPET), none of which differed according to baseline iron status. A differential effect of IV iron on maximal power output was detected, with the performance of ID individuals receiving iron improving. However, the effect size was only of the order of one percent, which is unlikely to be of clinical significance. Iron administration also increased the oxygen pulse ($\dot {\rm V}$o_2_ divided by heart rate) irrespective of iron status. The size of this effect was much larger, of the order of ten percent. There was a trend for IV iron to reduce minute ventilation ($\dot {\rm V}$E) at maximum power output irrespective of iron status. Data relating to lactate kinetics are presented in Table 5. Individuals randomised to receive IV iron showed an increase in lactate threshold of around ten percent regardless of baseline iron status. There was also a trend for IV iron to reduce peak lactate, again irrespective of iron status. No differential effect of IV iron was detected according to baseline iron status.

**Table 4:** Variables measured at the point of volitional fatigue during incremental CPET.

Iron status	Iron deficient (n = 13)				Iron replete (n = 13)				P-value (RM-ANOVA)
Intervention	Iron (n = 7)		Saline (n = 6)		Iron (n = 7)		Saline (n = 6)		
Visit	1	2	1	2	1	2	1	2	
Power, W	180 ± 49	181 ± 46	182 ± 52	178 ± 54	216 ± 42	212 ± 43	184 ± 45	186 ± 49	Iron status = 0.31 Visit & intervention = 0.62 **Iron status, visit & intervention = 0.027**
$\dot {\rm V}$o_2_, ml/kg	38.0 ± 11.3	38.0 ± 10.9	39.0 ± 8.2	36.1 ± 8.3	41.3 ± 7.7	41.4 ± 9.8	39.6 ± 4.7	39.3 ± 5.6	Iron status = 0.45 Visit & intervention = 0.10 Iron status, visit & intervention = 0.22
RER	1.15 ± 0.08	1.16 ± 0.09	1.14 ± 0.07	1.14 ± 0.06	1.15 ± 0.05	1.12 ± 0.02	1.12 ± 0.04	1.12 ± 0.06	Iron status = 0.40 Visit & intervention = 0.84 Iron status, visit & intervention = 0.33
$\dot {\rm V}$E, L/min	102 ± 42	98 ± 40	98 ± 21	100 ± 16	123 ± 33	121 ± 32	93 ± 26	96 ± 28	Iron status = 0.50 Visit & intervention = 0.11 Iron status, visit & intervention = 0.86
Oxygen pulse, ml/beat	12.4 ± 3.1	12.8 ± 3.1	13.0 ± 3.7	12.6 ± 3.5	14.9 ± 3.8	15.4 ± 3.9	13.1 ± 2.9	12.6 ± 2.8	Iron status = 0.34 **Visit & intervention = 0.036** Iron status, visit & intervention = 0.89
RPE	18.6 ± 1.5	18.3 ± 1.1	18.0 ± 1.8	17.8 ± 2.6	19.0 ± 1.0	18.9 ± 1.1	18.3 ± 1.0	18.5 ± 1.0	Iron status = 0.37 Visit & intervention = 0.61 Iron status, visit & intervention = 0.82

P-values are given for: (i) the effect of baseline iron status; (ii) the interaction between visit and intervention (the effect of IV iron); and (iii) the interaction between baseline iron status, visit, and intervention (whether any effect of IV iron differed according to baseline iron status). Values are means ± SD. Statistically significant P-values appear in bold. RER, respiratory exchange ratio; $\dot {\rm V}$E, minute ventilation; oxygen pulse is defined as $\dot {\rm V}$o_2_ divided by heart rate; RPE, rating of perceived exertion.

**Table 5:** Venous lactate kinetics during exhaustive exercise.

Iron status	Iron deficient (n = 13)				Iron replete (n = 13)				P-value (RM-ANOVA)		
Intervention	Iron (n = 7)		Saline (n = 6)		Iron (n = 7)		Saline (n = 6)		Iron status	Visit & intervention	Iron status, visit & intervention
Visit	1	2	1	2	1	2	1	2			
Peak lactate, mmol/L	6.2 ± 2.4	5.7 ± 1.9	6.4 ± 1.6	7.3 ± 2.3	6.7 ± 1.9	6.4 ± 2.3	6.3 ± 2.0	6.5 ± 2.3	0.90	0.12	0.46
$\dot {\rm V}$o_2_ lactate threshold, ml/kg	20.6 ± 6.9	23.0 ± 8.0	22.5 ± 6.9	22.7 ± 7.6	24.9 ± 6.3	28.0 ± 6.9	24.0 ± 4.7	24.0 ± 4.9	0.24	**0.039**	0.72

P-values are given for: (i) the effect of baseline iron status; (ii) the interaction between visit and intervention; and (iii) the interaction between baseline iron status, visit, and intervention. Values are means ± SD. Statistically significant P-values appear in bold. The peak lactate is that measured at volitional fatigue.

### Submaximal exercise

**Figure 2:**
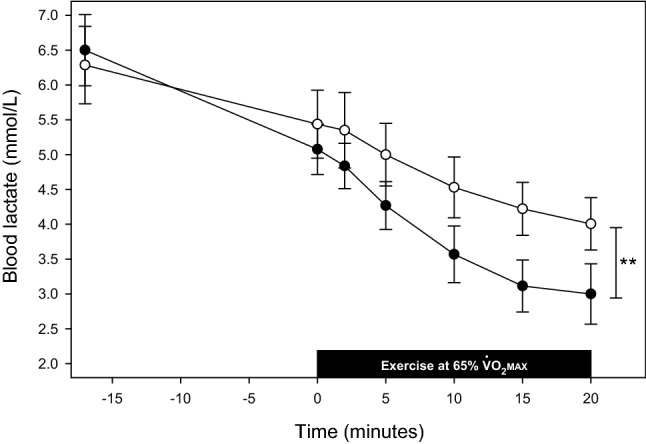
Blood lactate following volitional fatigue and during submaximal exercise at first study visit. Data for ID participants appear as white circles; those for IR participants, black circles. The initial venous lactate value was measured at volitional fatigue during the preceding maximal CPET. Following a 15-min interval, participants returned to the ergometer and measurements were made during two minutes seated at rest. The solid black bar indicates the period of submaximal exercise. A single lactate value was missing for one ID participant at the 2-min timepoint due to a technical issue. Values are means; error bars show SE. **, P = 0.005 for interaction of iron status and time.

Both groups exercised at very similar levels of $\dot {\rm V}$o_2_ throughout a subsequent 20-min period of submaximal exercise, close to the prescribed target of 65% $\dot {\rm V}$o_2_max (mean ± SD: ID group 67 ± 3.0%; IR group 66 ± 2.5%). At the first study visit, the only variable with grossly different behaviour between groups was venous lactate (Fig. 2). In a mixed effects model, lactate concentration fell significantly over the course of the rest and submaximal exercise period (P < 0.001 for time), but the rate of decline in lactate was slower in the ID group (P = 0.005 for interaction between time and iron status).

**Figure 3:**
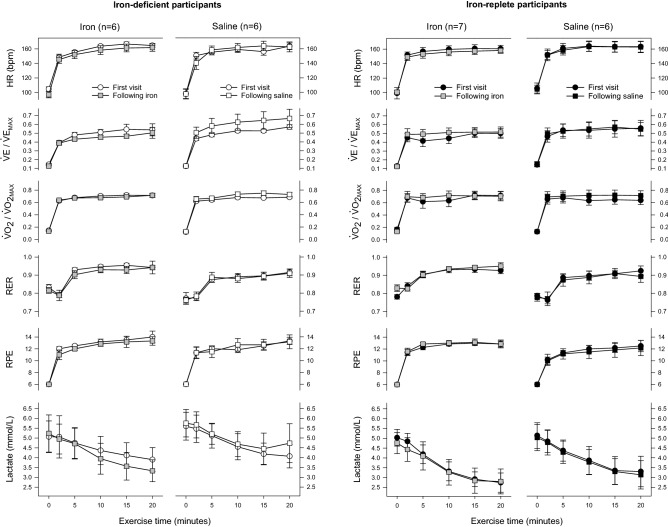
Cardiorespiratory variables during submaximal exercise at each study visit. Data for ID participants appear as white symbols; those for IR participants, black symbols. Data from the first visit appear as circles; those for the second, squares. Data for the second visit in participants receiving IV iron are shown in grey. $\dot {\rm V}$E and $\dot {\rm V}$o_2_ values for both visits are expressed relative to maximal values at the first visit. Values for parameters other than lactate are means for the previous 30 s; error bars show SE. One ID participant became presyncopal after volitional fatigue at the second visit and did not perform submaximal exercise; data for this individual are excluded from the figure (but not the statistical analysis). Data at the first visit for $\dot {\rm V}$E, $\dot {\rm V}$o_2_ and RER at the 10, 15 and 20-min time points were missing for one IR participant randomised to receive saline due to a technical issue. P = 0.028 for differential effect of IV iron according to baseline iron status; comparisons for all other variables NS. HR, heart rate; $\dot {\rm V}$E, minute ventilation; RER, respiratory exchange ratio; RPE, rating of perceived exertion.

For lactate kinetics pre- and post-infusion, there was a significant interaction between baseline iron status, intervention, visit, and time (Fig. 3). Specifically, IV iron had an effect to accelerate the fall in venous blood lactate only in the ID group (P = 0.028). Changes in other cardiorespiratory variables during submaximal exercise are also shown in Fig. 3.

### Skeletal muscle biopsy

Gene expression data from quantitative polymerase chain reaction (qPCR) of skeletal muscle sampled at the first study visit are given in Table 6. No significant differences were detected, although there is some suggestion that *LDHA* may have been higher in the ID group.

**Table 6:** Skeletal muscle mRNA expression levels at first visit.

Gene	ID group (n = 10)	IR group (n = 6)	P-value (t-test)
*LDHA*	1.51 ± 0.18	0.98 ± 0.12	0.06
*PFK*	1.15 ± 0.17	1.03 ± 0.15	0.63
*PDK1*	1.06 ± 0.13	1.07 ± 0.17	0.95

Expression data are means ± SE at the mRNA level for lactate dehydrogenase A (*LDHA*), phosphofructokinase (*PFK*), and pyruvate dehydrogenase kinase isoform 1 (*PDK1*).

## Discussion

### Main findings

The main finding of the present study is of abnormal whole-body metabolism in ID individuals, manifest as disturbed blood lactate kinetics during exercise, in the absence of any demonstrable impairment of skeletal muscle oxidative phosphorylation. Iron deficiency appears to promote a shift in favour of anaerobic glycolysis, reflected in sizeable changes in the threshold for anaerobic metabolism, and this effect is not mediated by differences in Hb concentration. Thus, the consequences of clinical iron deficiency in humans are seen to be similar with respect to lactate kinetics to the abnormalities reported in ID rodents^7,11^ and patients with CP^15^, without the gross disturbance of high-energy phosphate metabolism seen in these settings. To our knowledge, the present study is the first to describe an iron-mediated action of this sort in healthy humans, distinct from an effect of Hb concentration.

The rate of decline in blood lactate concentration was diminished in ID participants exercising at 65% $\dot {\rm V}$o_2_max. This implies that iron deficiency impairs lactate disposal, promotes its production, or causes a combination of these two phenomena. This effect cannot be explained by the marginally lower Hb concentration in the ID group, first, because an individually tailored submaximal work rate was employed for each participant, and second, because the apparent rate of lactate disappearance from the blood increased in ID participants in the absence of any increase in Hb concentration following IV iron. In further support of this view, at the first study visit there was a trend towards lower absolute lactate thresholds in ID participants compared with IR controls. Interestingly, administration of IV iron brought about a striking rise in lactate threshold measured during incremental CPET to volitional fatigue irrespective of baseline iron status, suggesting that the effects of iron status on lactate handling extend into what is considered the physiologically normal range, and are not limited solely to individuals that are iron deficient.

### Iron homeostasis

We measured a variety of indices of iron homeostasis over the course of the study. As expected, the ID and IR study entry criteria generated two groups that differed very significantly in iron status, with the mean values observed for serum ferritin and TSat in the ID group at enrolment consistent with depletion of storage iron to the point of absent stainable iron in the bone marrow^21,22^.

Ferritin is the major mammalian iron storage protein, serum levels of which correlate very strongly with mobilisable iron stores in health, with 1 µg/L serum ferritin corresponding to ~ 8 mg storage iron^23^. The mean increase in serum ferritin seen in those allocated to iron in the present study exceeded 500 µg/L in ID participants and approached 800 µg/L in the IR group. Given that no more than a 1000 mg dose of IV iron was administered to any participant, it can immediately be seen that these values indicate that soon after IV iron administration the utility of serum ferritin as a marker of storage iron is lost. The likely explanation is the regulation of ferritin mRNA translation by iron^24,25^, such that soon after IV infusion of a large iron dose, ferritin expression is greatly stimulated; this phenomenon has been observed in previous human physiology studies^26–28^. That the serum ferritin level achieved in ID participants given iron was lower in absolute terms than that seen in IR participants, suggests that pre-existing iron deficiency constrains ferritin expression by some means. This may be explained by the observation from radioactive isotope studies that iron is directed rapidly towards the bone marrow when infused into individuals with profound iron deficiency^29^.

Hepcidin is the major hormone regulating iron homeostasis, acting via the cellular iron exporter ferroportin to control intestinal iron absorption, the export of recycled iron from macrophages, and release of iron stored within hepatocytes^30^. Similar to the trend observed with ferritin, the magnitude of the rise in hepcidin when iron was administered was more substantial in the IR group than in ID participants. The regulation of hepcidin expression by iron is more complicated than ferritin, since it involves signals from developing erythrocytes as well as plasma iron concentration and body iron stores^28,30^. Nevertheless, our findings imply that existing tissue iron depletion acts as a strong negative regulatory signal for hepcidin expression, even when serum iron levels are acutely grossly elevated.

Finally, IV iron tended to suppress circulating erythropoietin levels in ID, but not IR, participants, implying that iron deficiency acts directly to augment erythropoietin expression, as the suppression occurred prior to any increase in Hb concentration. This finding confirms that the increase in circulating erythropoietin levels seen in heathy humans following iron chelation^31^ does have a clinical correlate, and is also in keeping with the demonstration in a mouse model that manipulation of iron availability alters the expression of the gene encoding erythropoietin by modulating levels of HIF-2α in renal fibroblasts^32^.

### Strengths and limitations

The main strengths of the present study are four-fold. First, the dual assessment in the same individuals of exercise metabolism using both MRS of a small muscle mass, and whole-body exercise. Second, the randomised use of a substantial dose of IV iron in a double-blind manner. Third, the recruitment of individuals with profound absolute iron deficiency in the absence of marked anaemia. Fourth, the repeat assessment of these individuals after IV iron but before any change in Hb concentration.

The dose of IV iron used was twice that employed in a contemporary study of elite athletes^9^ and brought about a striking change in circulating markers of iron homeostasis. With the exception of work in patients with CP^15^, no previous human study has to our knowledge used both CPET and MRS together in the same individuals, and most have been primarily concerned with athletic performance. To our knowledge, only two previous studies have used MRS to investigate the effects of iron status in humans. The first recruited a small, heterogeneous group of anaemic hospital inpatients^33^; the second studied patients with chronic heart failure and reported that iron deficiency was associated with more marked PCr depletion and profound intracellular acidosis with small muscle mass exercise, leading the authors to speculate that enhanced anaerobic glycolysis was occurring^34^. Neither whole-body exercise nor measurements of blood lactate were undertaken.

The limited reproducibility of MRS may have restricted our ability to detect a subtle effect of iron deficiency on skeletal muscle oxidative phosphorylation. When assessed using similar apparatus to that employed in the present study, very highly trained individuals were found to have generally reproducible measures of resting high-energy phosphorus metabolites, but the coefficient of variation during exercise for PCr was 27%, and that for PCr half-time, 40%^35^. Nevertheless, in healthy, non-trained individuals, PCr depletion during exercise appears to be much more highly reproducible, such that biological variability accounts for the vast majority of measurement variability^36^. Thus, whilst the present study was not powered to exclude a subtle impairment of skeletal muscle oxidative phosphorylation by iron deficiency, and we may have been unable to detect a small effect, gross abnormalities of mitochondrial function of the sort evident in CP patients^15^ and rodent models of iron deficiency^12^ are clearly absent.

We sampled venous, rather than arterial blood for real-time analysis of lactate concentration, as this was safer and more acceptable to participants. Measured venous blood lactate levels from antecubital fossa sampling have been shown to correlate closely with arterial values during lower limb exercise^37^, supporting such an approach. Haemoglobin concentration was slightly lower on enrolment in the ID group. In the case of the MRS data, the small muscle mass exercise used can reasonably be anticipated to be unaffected by any difference in systemic oxygen delivery^15^. Additionally, because participants were randomised to receive iron or saline, and no change in Hb concentration occurred between study visits, a haemoglobin-mediated effect can confidently be excluded.

It proved difficult to recruit ID males to the present study, such that recruitment fell short of the target sample size. The reasons are not entirely clear, since the same recruitment approach was effective in a previous study^38^. This should be acknowledged when considering the generalisability of the results. Also, we intentionally avoided recruiting only individuals habituated to aerobic exercise, unlike previous studies that have focused on iron status and athletic performance, so that our participants might better reflect the healthy general adult population. However, one consequence was that the distribution of values for $\dot {\rm V}$o_2_max and the anaerobic threshold within each group were rather broad.

Finally, we did not make skeletal muscle biopsy a compulsory part of the protocol because of its invasiveness. Instead, participants were free to decline the biopsy at the second visit having undergone it at the first, or not undergo biopsy at all. Comparisons of qPCR data between groups are therefore based on a subset of the participants at the first study visit; there were insufficient data from the second visit to assess the effect of IV iron.

### Underlying mechanisms

One mechanism historically proposed for the metabolic effects of iron deficiency is impaired function of oxidative enzymes requiring iron as a cofactor, particularly cytochrome C^11,39–43^. However, in rodent models, iron deficiency has typically been induced by severe early life dietary iron restriction, which tends to reduce concentrations of skeletal muscle myoglobin and iron-dependent mitochondrial enzymes^12^. These latter changes have not been demonstrated in adult humans with iron deficiency^44,45^, which may explain many of the differences between human iron deficiency and animal models thereof. Our ID participants arguably behaved in a Warburg-like manner; this is the opposite effect to that described in animals with tissue-specific deletion of skeletal muscle HIF-1α^46,47^, and in endurance athletes, who seem to exhibit downregulation of the HIF pathway^48^. Significant changes occur in skeletal muscle lactate production and disposal as a consequence of endurance training^49^, and it is possible that IV iron acted upon the HIF pathway in skeletal muscle mimicking these changes.

The rapid decay of HIF-1α when exposed to euoxic conditions^50^ precludes reliable direct measurement of levels of the protein itself in human studies in order to elucidate underlying mechanisms. However, the molecular biology of erythropoietin expression and its regulation by HIF are very well understood^17^, such that erythropoietin expression can be used from cell culture^51^ to intact healthy humans^31^ to study the effects of iron availability on the HIF pathway. The observed effects of iron supplementation on circulating erythropoietin levels in the present study are consistent with iron acting on the HIF-hydroxylases within renal fibroblasts^32^.

The site of action of iron deficiency with respect to lactate kinetics is less clear. The suggestion of increased *LDHA* expression in skeletal muscle of ID participants is of note, since resynthesis of glycogen within skeletal muscle seems to be a significant sink for lactate accumulated at the conclusion of strenuous exercise^52^. However, lactate is metabolised in significant quantities during exercise by both the heart and liver^53^, so alterations in cardiac or hepatic lactate handling may well underlie some of the observed effects in the present study. With respect to the latter possibility, HIF prolyl-hydroxylase 1 deficiency – which is the effective functional state that occurs as a result of iron deficiency – has been shown to increase glycolytic gene expression within the liver in a mouse model^54^.

The finding of an increased oxygen pulse at $\dot {\rm V}$o_2_max following IV iron infusion is of interest since the former is an index of stroke volume (SV) in healthy individuals (oxygen pulse = $\dot {\rm V}$o_2_/heart rate = SV × arteriovenous oxygen concentration difference)^55^. Since iron supplementation attenuates pulmonary vasoconstriction during both hypoxia^38,56,57^ and aerobic exercise^27^, another possibility is that an effect on stroke volume arises due to an action on the pulmonary circulation. The findings of a recent study involving IV iron supplementation prior to ascent to very high altitude would support this possibility^26^. Alternatively, increased tissue oxygen extraction might occur following iron supplementation. It is not possible from an integrated study to determine the relative contribution of these mechanisms.

### Clinical implications

Iron deficiency is associated with worse outcomes in chronic health conditions that are extremely prevalent globally, including chronic obstructive pulmonary disease^58^ and a number of chronic cardiovascular diseases^59^. Studies have indicated that intravenous iron supplementation can have beneficial effects in such conditions^60,61^. Of note, one study of patients with idiopathic pulmonary arterial hypertension reported a rise in anaerobic threshold following IV iron along with increased submaximal exercise endurance^62^, reminiscent of the present study. Importantly, impaired submaximal exercise performance is arguably most functionally relevant for patients with cardiopulmonary disease^53^, reflecting as it does the impact these conditions can have on everyday life. On the other hand, $\dot {\rm V}$o_2_max is an indicator of cardiopulmonary fitness that predicts outcomes, for example, following major surgical procedures^63^.

The present study furthers our understanding of the mechanisms by which iron deficiency is injurious to human health, and suggests that functional performance might be affected by IV iron in a broader range of conditions than has previously been recognised.

## Methods

This was a prospective, case–control, clinical physiology study with subsequent double-blind randomisation. By targeting blood donors, who were offered study information if below the Hb concentration threshold when attending to donate, we enrolled adults with absolute iron deficiency (serum ferritin ≤ 15 ug/L with TSat < 16%)^38^. Healthy age- and sex-matched IR volunteers (serum ferritin ≥ 20 ug/L with TSat ≥ 20%) served as controls. A screening visit was conducted consisting of medical history, physical examination, spirometry (MicroLab™, CareFusion, UK), and venous bloods. Those with factors likely to disturb cardiopulmonary physiology or iron metabolism were excluded, for example chronic inflammatory disorders, chronic lung disease, haemochromatosis, recent iron supplementation or blood transfusion, and recent long-haul air travel or residence at high altitude. Eligible participants attended two identical study visits approximately a week apart, commencing early in the morning following an overnight fast.

### Exercise protocol

Skeletal muscle ^31^P-MRS was performed using a 3 Tesla MRI scanner (Siemens TIM Trio) with the participant supine and a dual-tuned ^31^P and ^1^H 6-cm-diameter surface coil secured under the right calf. The foot was fastened to a custom-built plantarflexion exercise apparatus with the leg straightened, and the calf exercised at 1 Hz in time to a digital metronome, as previously described^15^. Three 5-min periods of exercise, at 3 W, 4 W and 5 W alternated with 7-min recovery periods. Data were processed offline by a blinded investigator.

Following MRS, a 20-gauge venous cannula (Venflon™, Becton Dickinson) was inserted into a large antecubital fossa vein and blood drawn. Incremental CPET to volitional fatigue was performed on an electronically braked cycle ergometer (ergoselect 100, ergoline GmbH, Germany). Participants wore a close-fitting facemask with respired gases sampled continuously through a catheter and analysed by indirect calorimetry (Metalyzer^®^ 3B CPET System, CORTEX Biophysik GmbH, Germany). Resting data were collected over a 2-min period. Work then began at 50 W, for three minutes, increasing thereafter by 25-W increments at 3-min intervals. Venous blood was sampled via the indwelling cannula during the final 30 s of exercise at each workload and upon cessation of exercise. Samples were analysed using a clinically validated Lactate Pro™ device (Arkray Inc. Japan)^64^. Participants rated their perception of exertion on a fifteen-point RPE scale, from 6 (rest) to 20 (maximum exertion)^65^.

After a rest period of 15 min following volitional fatigue, participants returned to the ergometer and 2 min of resting data were recorded. There then followed a further 20 min of exercise at a work rate equivalent to 65% $\dot {\rm V}$o_2_max, determined by interpolation of the data just obtained during maximal CPET. During submaximal exercise, blood was sampled at rest, after 2 min, 5 min and every 5 min thereafter. On the second study visit, the same work rate was used during submaximal exercise. Exercise data were processed offline by an investigator blinded to participant iron status and randomisation. The lactate threshold was determined as described by Beaver and colleagues^66^.

### ^31^P magnetic resonance spectroscopy data processing

^31^P-MRS spectral peaks were fitted using the automated AMARES algorithm within the jMRUI software package, with absolute concentrations of phosphorus metabolites calculated from the spectral data using an established method^15,67–69^. PCr recovery kinetics were modelled for the period after each exercise bout. A monoexponential relationship was derived^70^, using a least-squares-fit approach to determine the time-constant (τ), and expressing [PCr] as a function of time (t)^71^:

$$\left[ {{\text{PCr}}} \right]_{{\text{t}}} = \, \left[ {{\text{PCr}}} \right]_{0} + \, \left( {\left[ {{\text{PCr}}} \right]_{{{\text{Rest}}}} - \, \left[ {{\text{PCr}}} \right]_{0} } \right) \cdot \left( {1 \, {-}e^{{( - {\text{t}}/\uptau )}} } \right)$$

The maximum theoretical rate of mitochondrial ATP synthesis (Qmax) was extrapolated from a combination of the end-exercise [ADP] and initial rate of PCr resynthesis, as follows:

$${\text{Q}\textsc{max}} = {\text{V}} \cdot (1 + ({\text{K}}_{{\text{m}}} \div \left[{\text{ADP}}\right])^{\text{n}})$$

where ${\rm V}$ is the initial rate of PCr resynthesis, K_m_ is the [ADP] at which oxidative ATP synthesis is taken to be half maximal (25 µmol/L) and n (2.2) is a Hill coefficient that describes the relationship between ${\rm V}$ and [ADP]^35,72,73^.

### Skeletal muscle biopsy and qPCR

Prior to maximal CPET, participants agreeing to undergo skeletal muscle biopsy reclined comfortably on an examination couch with their right leg fully relaxed. The lateral aspect of the thigh was cleaned with 0.5% chlorhexidine in 70% ethanol, and local anaesthesia procured with ~ 5 ml 1% lidocaine infiltrated using a 22-gauge needle. An 11-blade surgical scalpel was used to penetrate the skin and breach the superficial fascia. A disposable sterile spring-loaded core biopsy instrument (Monopty^®^, Bard Peripheral Vascular Inc. Tempe, AZ) was inserted through the incision and discharged to take a sample of skeletal muscle, which was immediately flash-frozen in liquid nitrogen. Four passes were made in total. Haemostasis was ensured and a sterile dressing applied.

Total RNA was extracted from 20–30 mg powdered skeletal muscle using the RNeasy^®^ Fibrous Kit (Qiagen, UK). The crushed tissue underwent tissue rupture on ice followed by a DNase treatment step, before complementary DNA was synthesized from 1 μg RNA using the Applied Biosystems High Capacity cDNA Reverse Transcription Kit (Life Technologies, UK). Real-time PCR was performed using an ABI StepOnePlus™ Real-Time PCR System (Applied Biosystems, UK) with TaqMan^®^ Universal PCR Master Mix and TaqMan Gene Expression Assays (choosing manufacturer-recommended assays: Applied Biosystems, UK). Relative mRNA expression levels were determined using the standard curve method and normalised to beta-actin.

### Venous blood analyses and infusions

Assays for full blood count, C-reactive protein (CRP), serum ferritin, iron, transferrin and TSat were performed by a University Hospital clinical pathology laboratory. Aliquots of serum and EDTA plasma were obtained by centrifugation and frozen and stored at − 80 °C until the conclusion of the final study visit of the final participant. Serum erythropoietin, plasma sTfR (both Quantikine^®^, R&D Systems, Abingdon, UK), and plasma hepcidin (Hepcidin 25 HS, DRG, Marburg, Germany) were analysed by enzyme-linked immunosorbent assay. At the end of the first study visit, participants received either ferric carboxymaltose (Ferinject^®^, Vifor Pharma, Switzerland) 15 mg/kg (maximum 1 g) or 0.9% saline. Block randomisation according to iron status and sex was used. Participants were blindfolded, and the infusion, infusion line and infusion site obscured using an opaque plastic drape.

### Study approval

The study received ethical approval from the NHS South Central Oxford B Research Ethics Committee (reference 13/SC/0439). The study was sponsored by the University of Oxford and conducted in accordance with the principles of the declaration of Helsinki. All participants gave written informed consent. The study was registered with ClinicalTrials.gov (NCT02308449).

### Statistics

To estimate the sample size required we considered: (i) work in CP patients showing a 1.6 to 1.9 fold greater fall in PCr during calf muscle exercise compared with healthy individuals^15^; (ii) data from individuals with IDA in whom the ratio of PCr to (PCr + P_i_) fell approximately one third more than in healthy individuals during small muscle mass exercise^33^; and (iii) evidence that the degree of PCr depletion during exercise in non-trained individuals is highly reproducible when measured with with MRS^36^. We calculated that we would need to study a total of 32 volunteers in order to have 80% power to detect 15% greater PCr depletion in ID compared with IR individuals, with a significance level of P < 0.05.

Data were analysed using SPSS Statistics (version 25, IBM). The two-sided unpaired Student’s t-test and Mann Whitney U test were used for group comparisons. Repeated measures analysis of variance (RM-ANOVA) and mixed-effects modelling were used to determine the effects of exercise and iron infusion within and between groups.

## Data Availability

The data that support the findings of this study are available on request from the corresponding author. The data are not publicly available due to privacy or ethical restrictions.
