# Publisher Correction: Abnormal whole-body energy metabolism in iron-deficient humans despite preserved skeletal muscle oxidative phosphorylation

**DOI:** 10.1038/s41598-022-06694-7

**Published:** 2022-03-01

**Authors:** Matthew C. Frise, David A. Holdsworth, Andrew W. Johnson, Yu Jin Chung, M. Kate Curtis, Pete J. Cox, Kieran Clarke, Damian J. Tyler, David J. Roberts, Peter J. Ratcliffe, Keith L. Dorrington, Peter A. Robbins

**Affiliations:** 1 Department of Physiology, Anatomy and Genetics, University of Oxford, Sherrington Building, Parks Road, Oxford, OX1 3PT UK; 2 Nuffield Department of Clinical Neurosciences, University of Oxford, John Radcliffe Hospital, Oxford, OX3 9DU UK; 3 Nuffield Department of Clinical Laboratory Sciences, National Blood Service Oxford Centre, University of Oxford, John Radcliffe Hospital, Oxford, OX3 9BQ UK; 4 Nuffield Department of Medicine, University of Oxford, NDM Research Building, Old Road Campus, Headington, OX3 7FZ Oxford UK; 5 Francis Crick Institute, London, NW1 1AT UK

Correction to: *Scientific Reports* 10.1038/s41598-021-03968-4, published online 19 January 2022

The original version of this Article contained a repeated error where the ‘${\dot{\mathrm{V}}}$’ symbol did not display correctly in the Introduction, the Results section under the subheadings ‘Exercise to volitional fatigue’ and ‘Submaximal exercise’, the Discussion section under the subheadings ‘Main findings’, ‘Strengths and limitations’, ‘Underlying mechanisms’ and ‘Clinical implications’, the Methods section under the subheading ‘Exercise protocol’, Table 4 and 5 and the legends of Figure 3 and Table 4.

As a result,

“$${\dot{\mathrm{V}}}$$**˙**o_2_max”.

now reads:

“$${\dot{\mathrm{V}}}$$o_2_max”.

“$${\dot{\mathrm{V}}}$$**˙**E”.

now reads:

“$${\dot{\mathrm{V}}}$$E”.

“$$\mathop {{\text{V}}^{ \cdot } }\limits^{  \cdot}$$o_2_”.

now reads:

“$${\dot{\mathrm{V}}}$$o_2_”.

Furthermore, in the Methods section, under the subheading ‘^31^P magnetic resonance spectroscopy data processing,’

“where ${\dot{\mathrm{V}}}$ is the initial rate of PCr resynthesis, K_m_ is the [ADP] at which oxidative ATP synthesis is taken to be half maximal (25 µmol/L) and n (2.2) is a Hill coefficient that describes the relationship between ${\dot{\mathrm{V}}}$ and [ADP]^35,72,73^.”

now reads:

“where V is the initial rate of PCr resynthesis, K_m_ is the [ADP] at which oxidative ATP synthesis is taken to be half maximal (25 µmol/L) and n (2.2) is a Hill coefficient that describes the relationship between V and [ADP]^35,72,73^.”

In Table 4, in the 1^st^ column ‘Visit’,

“$${\dot{\mathrm{V}}}$$**˙**E (L/min)”.

now reads:

“$${\dot{\mathrm{V}}}$$E (L/min)”.

The original Article has been corrected.

